# Custom Metal Occlusal Surface for Acrylic Resin Denture Teeth to Enhance Wear Resistance: A Case Report

**DOI:** 10.1155/2012/264620

**Published:** 2012-09-10

**Authors:** Rizwan Ali Shivji, Vaibhav D. Kamble, Mohd. Atif Khan

**Affiliations:** Department of Prosthodontics, VSPM Dental College and Research Centre, Nagpur, India

## Abstract

Wear of the occlusal surface of the denture is a known fact which leads to subsequent changes in jaw relation, vertical dimension, loss of aesthetics, aged looks, and decrease in masticatory efficiency. Treatment modalities includes, change of denture set after a regular interval of 4-5 years, use of wear resistant denture teeth that includes wear resistant resin or porcelain teeth, teeth with cast metal occlusal surface, and altering occlusal contact areas of denture teeth by use of silver amalgam fillings. A case report of a patient who had increased tendency of occlusal wear was treated with custom made metal occlusal surface of denture teeth to enhance wear resistance and to improve the masticatory efficiency.

## 1. Introduction

Thought is the child of action and necessity is the mother of invention. This is the soul of newly based dentistry. A little diverse approach from traditional modalities available in dentistry can solve patient's problem to a greater extent, thereby adding to his well-being and comfort. Wear changes in the occlusal surface of the denture wearing patient is a known fact. But need to change the dentures on an increased frequency ask a call for lateral thinking. This calls the change in approach and technique. One such patient reported to VSPM Dental College and Research Centre, Nagpur, who had frequent need for change of his denture sets due to excessive wear, resulting in loss of masticatory efficiency, function, aesthetics, and comfort.

## 2. Case Report

**Figure 1:**
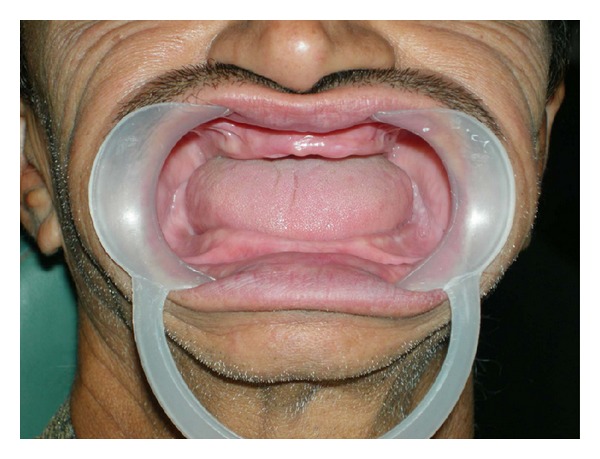
Preoperative view: complete edentulous arches.

**Figure 2:**
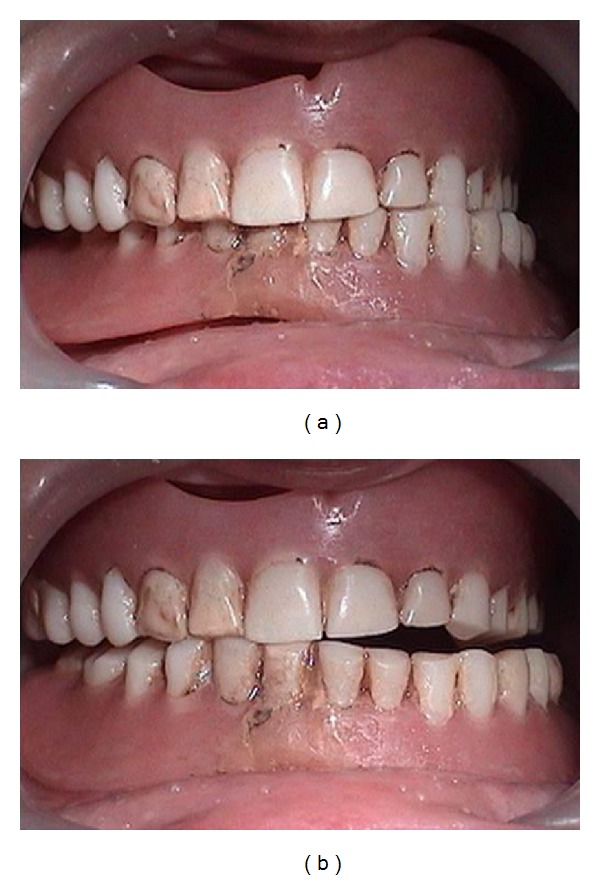
Wear of prosthetic denture teeth leading to difficulty in mastication.

A 58-years-old male patient reported in the Department of Prosthetic Dentistry, VSPM Dental College and Research Centre, Nagpur with the chief complaint of worn out denture occlusal surfaces and difficulty in mastication (Figure 1). Detailed medical and dental history revealed that the patient was suffering from neuromuscular disorder (differential diagnosis-trigeminal myoclonus) resulting in repetitive episodes of cyclic, involuntary, and uncoordinated brisk jaw movements. Oral manifestations showed severe wear of occlusal surface of prosthetic teeth (Figures 2(a) and 2(b)). Further it was found that it was his fourth denture set within a span of three years. Although the retention of the denture was satisfactory, he was forced to have his denture remade frequently. This forced the patient to undergo great physical and mental stress of dental procedures and frequent visits to dental operatory. To meet this vague problem and to reduce wear rate of prosthetic denture teeth, it was decided to fabricate the complete denture prostheses with cast metal occlusal surface.

**Figure 3:**
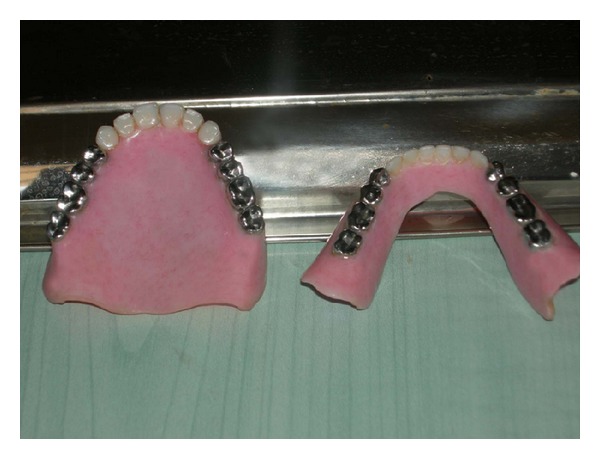
Custom metal occlusal surfaces for acrylic resin denture teeth.

**Figure 4:**
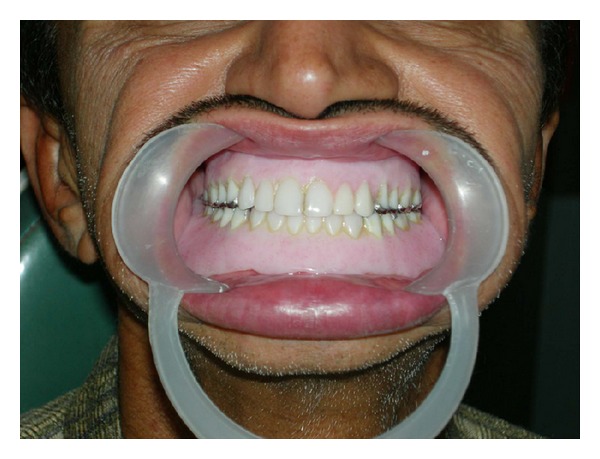
Postoperative intraoral view: denture insertion with metal occlusal surfaces on posterior teeth.

**Figure 5:**
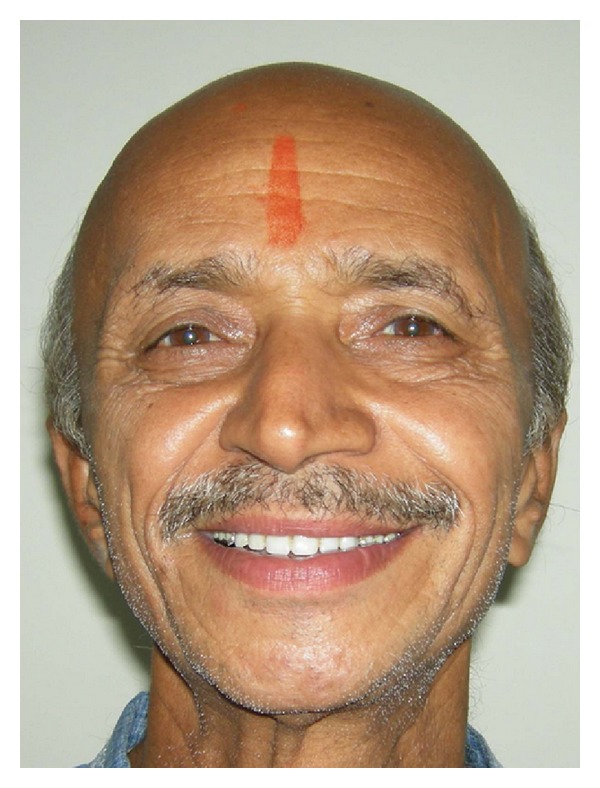
Postoperative extraoral view.

Metal occlusal surface are used to construct denture that oppose natural dentition or for reconstruction of dental arch with metal occlusal surface. Wallace [1] used gold for occlusal surface. On comparing the wear of gold, porcelain, heat-cured, and light-cured resin in occlusal contact, Ekfeldt and Oilo [2] found that all materials had the greatest loss of substance when the opposing teeth were of porcelain. The heat-cured, unfilled resin was the least wear-resistant material, followed by light-cured resin, porcelain, and gold. The heat-cured resin showed a combined tribochemical and fatigue type of wear. The light-cured resin and porcelain showed mainly a fatigue type of wear, whereas gold showed a combined abrasive and fatigue type of wear. Tanaka et al. [3] stated that the traditional denture fabrication technique using resin teeth with metal occlusal surfaces is rather complicated and time consuming. The teeth are arranged on the wax denture and processed in the usual manner. The patient is allowed to use the completed denture for a period of time to harmonize the occlusal surfaces with oral function. The occlusal portions of the teeth are then separated from the base and invested as a resin pattern for casting. The new cast occlusal portions are reset on the base portions with a resin adhesive. Krantz et al. [4] described a simplified method for making esthetic cast metal occlusal surfaces. Posterior acrylic resin teeth casted in a nickel-chrome alloy are coated with silane and an esthetic composite resin veneer is applied to the buccal surface. These veneered posterior metal teeth are incorporated in the wax setup and the dentures are processed and finished. Ekfeldt et al. [5] conducted a study to compare a gravimetric method and an impression technique in the evaluation of occlusal substance loss. The wear of gold, porcelain, and microfilled resin was studied in vivo. The gravimetric method showed lower substance loss for porcelain than for gold, whereas the microfilled resin had the highest substance loss. The observed findings corroborated with previous findings of the wear mechanism of the materials; that is, gold has mainly abrasive wear in contact with porcelain, whereas porcelain has a fatigue type and microfilled resin a tribochemical type of wear. After complete evaluation of the patient's problem and need, a modified technique of fabricating denture prosthesis with metal occlusal surface was planned. Standard technique using conventional clinical and laboratory steps were followed to fabricate the denture and essential modifications were made to attend anticipated results. Following satisfactory wax try-in appointment, Putty index impression of denture teeth were made for each quadrant separately with polyvinyl siloxane impression material (Aquasil Soft putty/Regular set, Dentsply, Germany). Wax pattern from the same were made using inlay casting wax (Harvard, Blauwachs, Berlin, Germany) with attention to get occlusal and lingual/palatal surface anatomy of each quadrant. Wax patterns were further refined and retentive tags and beads were added for retention of the tooth coloured acrylic facing and also for mechanical locking of the prosthetic tooth to denture base. Patterns were invested in phosphate bonded investment (Bellasun, Bego, Germany) and casted in nickel-chromium alloy (Ruby Dental Products Inc., Osaka, Japan). Castings were finished and polished using standard procedures. Acrylic resin facings were then added to the castings with appropriate tooth coloured heat polymerized acrylic resin (A3) (DPI, Mumbai, India). Acrylic denture teeth were replaced with custom-made metal occlusal surface denture teeth in respective quadrants maintaining the verified jaw relation record on the articulator. Wax try-in was repeated for final evaluation of the jaw relation, phonetics, aesthetics, and occlusion. Prosthesis was processed, finished, and polished following laboratory remount procedure (Figure 3). Denture insertion was done following the necessary clinical remount procedure (Figure 4). Recall visits were scheduled after 24 hours, 1 week, 1 month, and then every subsequent 3 months initially. Patient was on regular yearly follow up thereafter. Denture provided the effective services to the patient satisfaction (Figure 5). Seeing the patient's compliance and complete satisfaction of denture prosthesis, new dentures were fabricated using the same procedure after six years.

## 3. Discussion

The use of standard technique with required indicated clinical and laboratory alterations may contribute to greater clinical success. With this technique there is increase in one clinical and laboratory step, but had contributed significantly in solving patient's long term problem. Metal occlusal surface has advantages of inherent physical property of metal, the adaptability of the occlusal surface and a definite albeit subjective and psychological advantage. Disadvantages of metal occlusal surface include compromised aesthetics, mechanical method of locking of the acrylic resin and increased weight of prosthesis. Alternative to metal occlusal surface, porcelain teeth can be used but they contribute to maximum stress, brittle, expensive, and mechanical locking with denture base [2, 5]. Silver amalgam filling may be used but will cover only occlusal fossae, pit and fissure areas but not the complete occlusal surface. This will be mostly effective in cases with single complete denture but the advantages are economical and require less clinical and laboratory steps.

## 4. Conclusion

In a nut shell, the present approach towards the patient's diverse problem has served as a better treatment modality. The approach seemed to be best suited for the patients not too keen on posterior aesthetics. Patients opinion and compliance is been taken annually since 4 years of denture use. Patient seemed to be happy and satisfied with the denture service with no need for remake to be done since then.
